# 阿扎胞苷联合高三尖杉酯碱通过调控c-MYC/DDIT3/PUMA轴协同抗急性髓系白血病的研究

**DOI:** 10.3760/cma.j.issn.0253-2727.2023.12.006

**Published:** 2023-12

**Authors:** 军 李, 炎青 黄, 杰 訾, Chunhua Song, 峥 葛

**Affiliations:** 1 东南大学附属中大医院血液科，东南大学血液病研究所，南京 210009 Department of Hematology, Zhongda Hospital, School of Medicine, Southeast University, Institute of Hematology Southeast University, Nanjing 210009, China; 2 Pennysvinia State University, College of Medicine and Hershey Medical Center, Hershey, PA 17033, USA; 3 Division of Hematology, The Ohio State University Wexner Medical Center and The James Cancer Hospital, Columbus, OH 43210, USA

**Keywords:** 阿扎胞苷, 高三尖杉酯碱, 白血病，髓系，急性, 协同效应, Azacitidine, Homoharringtonine, Leukemia, myeloid, acute, Synergistic effect

## Abstract

**目的:**

探索阿扎胞苷（AZA）联合高三尖杉酯碱（HHT）治疗急性髓系白血病（AML）的协同效应及其分子机制。

**方法:**

采用细胞增殖、凋亡和克隆形成实验研究AZA联合HHT在AML中协同效应并计算两药协同效应指数（CI），通过转录组测序、通路抑制剂和基因敲降等方法，探索两药的协同机制。

**结果:**

与单药相比，AZA+ HHT可显著抑制AML细胞增殖，并具有显著的协同效应（U937、MV4-11和KG-1细胞中CI值均小于0.9）；AZA+HHT能显著抑制U937（*P*<0.001）和MV4-11（*P*<0.001）细胞的克隆形成，并显著促进U937（*P*<0.001）和MV4-11（*P*<0.001）细胞凋亡。AZA联合HHT通过激活整合应激反应（ISR）信号通路介导DDIT3-PUMA依赖的AML细胞凋亡。AZA联合HHT可明显下调c-MYC蛋白，通过激活ISR信号通路，调控 c-MYC/DDIT3/PUMA轴，促进细胞凋亡发挥协同抗AML作用。

**结论:**

AZA联合HHT通过激活ISR信号通路，调控c-MYC/DDIT3/PUMA轴，抑制细胞增殖和促进细胞凋亡，发挥协同抗AML作用。

## Abstract (en)

**Objective:**

This study aimed to explore the synergistic effect and underlying mechanism of azacitidine（AZA）in combination with homoharringtonine（HHT）in acute myeloid leukemia（AML）.

**Methods:**

The synergistic effects of AZA and HHT were examined by cell proliferation, apoptosis, and colony formation assays. The synergistic effects were calculated using the combination index（CI）, and the underlying mechanisms were explored using RNA sequencing, pathway inhibitors, and gene knockdown approaches.

**Results:**

Compared with the single-drug controls, AZA and HHT combination significantly induced cell proliferation arrest and showed a synergistic effect with CI <0.9 in AML cells. In the combination group versus the single-drug controls, colony formation was significantly decreased, whereas apoptosis was significantly increased in U937（*P*<0.001）and MV4-11（*P*<0.001）cells. AZA and HHT combination activated the integrated stress response（ISR）signaling pathway and induced DDIT3-PUMA-dependent apoptosis in cells. Furthermore, it remarkably downregulated the expression of c-MYC. The combination also activated c-MYC/DDIT3/PUMA-mediated ISR signaling to induce synergy on apoptosis. The synergy of AZA+HHT on apoptosis was induced by activating c-MYC/DDIT3/PUMA-mediated ISR signaling.

**Conclusion:**

The combination of AZA and HHT exerts synergistic anti-AML effects by inhibiting cellular proliferation and promoting apoptosis through activation of the ISR signaling pathway via the c-MYC/DDIT3/PUMA axis.

急性髓系白血病（AML）是由造血祖细胞的恶性克隆和细胞分化阻滞而导致的一组异质性的血液恶性肿瘤[1]。目前AML标准诱导方案为“3+7”（蒽环类药物联合阿糖胞苷）[2]。近年来，不少研究探索新的联合方案，特别是针对老年或不能耐受强烈化疗的AML患者[3]–[7]。

阿扎胞苷（AZA）是一种去甲基化药物，通过DNA去甲基化和细胞毒作用发挥抗癌作用[8]。AZA联合不同药物治疗AML是近年研究热点，如AZA联合米哚妥林[5]、来那度胺[6]、度伐利尤单抗[7]、吉妥珠单抗[9]及维奈克拉[3]等。高三尖杉酯碱（HHT）是一种天然植物生物碱，通过抑制JAK2-STAT5 [10]、NF-κB信号通路[11]等发挥抗白血病作用。既往报道以HHT为基础方案治疗AML有较好疗效[12]。

近期，我们的临床研究（NCT04248595）结果显示AZA联合HHT为基础方案治疗老年/不能耐受化疗（AZA+HAG方案）、年轻/能耐受化疗（AZA+HIA方案）患者均取得较好疗效[13]–[14]。为进一步深入探究其理论基础，本研究拟探索AZA联合HHT抗AML细胞的协同作用及其潜在分子机制。

## 材料与方法

1.细胞系和试剂、器材：AML细胞系U937、MV4-11和KG-1来源于自美国ATCC细胞库，细胞培养于含10％胎牛血清（FBS）的RPMI 1640或IMDM（KG-1：20％FBS）培养基，在5％ CO_2_、37 °C的条件下培养。培养基及血清购自美国Gibco公司。

HHT为中国民生药业集团有限公司产品；AZA为美国Apexbio公司产品；ISRIB（NO.SC4332）、Z-VAD-fmk（NO.SC4332）为碧云天生物技术有限公司产品；Cell Counting Kit-8（CCK-8）试剂购自上海东仁化学科技有限公司；MTT（M1025）为索莱宝生物科技有限公司产品，细胞凋亡试剂盒（NO.550474、NO.550474、NO.559925）为美国BD公司产品；甲基纤维素培养基（H4100）为美国Stemcell公司产品；TRIzol试剂、荧光定量PCR试剂盒为日本TaKaRa公司产品；lentiviral的质粒载体（PLV3ltr-ZsGreen-Puro-U6）、包装质粒（pMD2.0、psPAX2）为南京科瑞斯生物公司产品；Exfect转染试剂（TD101）为诺维赞生物科技有限公司产品；嘌呤霉素（CAS.58-58-2）为美国InvivoGen公司产品；Bax（No.ab32503）、BCL2（No.ab32124）、c-MYC（No.ab32072）抗体为英国Abcam公司产品；cleaved caspase 3（No.9661）、ATF4（No.11815）、DDIT3（No.2985）、PUMA（No.12450）、eIF2α（No.9722）、p-eIF2α（No.3398）抗体为美国Cell Signaling Technology公司产品；GAPDH抗体（NO.1E6D9）为美国Proteintech公司产品。

实验器材紫外分光光度计（Multiskan Go）、实时定量PCR仪（StepOne Plus）、流式细胞仪（Attune NxT）均为美国Thermo公司产品，化学发光呈像仪（iBright）为美国Invitrogen公司产品。

2. CCK-8 细胞增殖实验：细胞以1×10^4^/孔的密度种植于96孔板中。将不同浓度的AZA（U937细胞：0、0.25、0.5、1、1.25、1.5、2、2.5 µmol/L；MV4-11细胞：0、0.0625、0.125、0.25、0.5、1、2、4 µmol/L；KG-1细胞：0、0.15、0.30、0.6、1.25、2.5、5 µmol/L）和HHT（U937细胞：0、10、20、30、40、50、60、100 nmol/L；MV4-11细胞：0、2.5、5、10、15、20、25、30、50、60 nmol/L；KG-1细胞：0、12.5、25、50、75、100、150 nmol/L）处理AML细胞48 h后，向每孔中加入10 µl CCK-8，37 °C孵育4 h。用紫外分光光度计测定吸光度（*A*）值并计算药物介导的细胞增殖抑制率。

3. 克隆形成实验：AML细胞在AZA和（或）HHT预处理12 h后离心收集并洗涤细胞，然后将细胞以500个存活细胞/孔的密度接种在甲基纤维素培养基中，10～14 d后用MTT（5 mg/ml）染色，计数克隆形成单位（CFU）。CFU百分比＝实验组CFU/对照组CFU×100％。

4. 细胞凋亡检测：细胞经药物处理48 h后离心收集，Annexin Ⅴ-FITC和PI染色，GFP（+）细胞用Annexin Ⅴ-APC和7-AAD抗体染色后进行后续流式细胞术分析。

5. RNA分离和实时定量RT-PCR（RQ-PCR）：应用TRIzol从细胞中分离出总RNA，用Superscript First-Strand Synthesis System从1 µg总RNA中生成cDNA。应用实时定量PCR仪进行RQ-PCR。基因的相对表达值由2^−ΔΔCt^方法确定。RQ-PCR的引物序列见表1。

**表1:** 实时定量PCR的引物序列

基因名称	上游引物	下游引物
DDIT3	5′-GAGAATGAAAGGAAAGTGGCAC-3′	5′-ATTCACCATTCGGTCAATCAGA-3′
PMAIP1	5′-GCAGAGCTGGAAGTCGAGTGTG-3′	5′-AGGTTCCTGAGCAGAAGAGTTTGG-3′
GAPDH	5′-GCAAATTCCATGGCACCGT-3′	5′-GACTCCACGACGTACTCAGC-3′

6. 全转录组测序分析：样品制备按照测序公司（诺禾致源公司）要求进行，依次进行Poly（A）抓取（mRNA富集）、双链cDNA合成、末端修复、接头连接、片段分选、PCR扩增和产物质检等过程，完成文库构建，上机测序，测序平台为Illumina HiSeq 2500。

7. 质粒转染、慢病毒载体构建及靶基因敲降：靶基因短发夹RNA（shRNA）质粒和包装质粒通过转染试剂转染293T 细胞后产生Lentivirus并纯化。Lentivirus感染AML细胞 72 h后，用嘌呤霉素筛选稳定转染株。对GFP 阳性率>95％的细胞进行蛋白检测和进一步功能分析。靶基因shRNA 寡核苷酸序列见表2。shCTL组代表转染了空载质粒的对照组。

**表2:** PUMA、DDIT3和c-MYC的shRNA的Oligo引物序列

基因名称	寡核苷酸设计
PUMA-iF	gatccGGTCCTGTACAATCTCATCATtcaagagATGATGAGATTGTACAGGACCtttttt
PUMA-iR	aattaaaaaaGGTCCTGTACAATCTCATCATctcttgaATGATGAGATTGTACAGGACCg
DDIT3-iF	gatccGACTGATCCAACTGCAGAGATtcaagagATCTCTGCAGTTGGATCAGTCtttttt
DDIT3-iR	aattaaaaaaGACTGATCCAACTGCAGAGATctcttgaATCTCTGCAGTTGGATCAGTCg
c-MYC-iF	gatccGCTTCACCAACAGGAACTATGtcaagagCATAGTTCCTGTTGGTGAAGCtttttt
c-MYC-iR	aattaaaaaaGCTTCACCAACAGGAACTATGctcttgaCATAGTTCCTGTTGGTGAAGCg

8. Western blot法检测蛋白表达水平：离心收集细胞，应用柱式蛋白提取试剂盒提取蛋白样本，加入SDS上样缓冲液后煮沸变性蛋白样本，然后进行电泳、转膜、封闭，再依次进行一抗、二抗孵育，最后使用化学发光成像仪显影检测蛋白表达。在检测药物处理对eIF2α、磷酸化eIF2α（p-eIF2α）、ATF4、DDIT3、PUMA表达的影响时，用40 µmol/L Z-VAD-fmk（pan-caspase抑制剂）预处理细胞1 h以抑制半胱氨酸蛋白酶的活化。

9. 统计学处理：采用GraphPad Prism 8.0进行统计学分析，数据以均值±标准差表示。组间比较采用*t*检验。采用Calcusyn软件计算协同效应指数（CI）（CI<0.9表示有协同作用，<0.7表示有强协同作用）。*P*<0.05为差异有统计学意义。

## 结果

1. AZA联合HHT协同抑制AML细胞增殖并促进细胞凋亡：分别在U937、MV4-11和KG-1细胞中，应用不同剂量AZA或HHT处理AML细胞48 h，研究AZA或HHT单药对AML细胞增殖的影响，结果显示AZA或HHT单药对以上3种AML细胞均呈剂量依赖的细胞毒性作用。联合作用分析显示，AZA+HHT对U937（图1A）、MV4-11（图1B）和KG-1细胞增殖均有抑制作用，在三株AML细胞中的CI值均<0.9，表明有协同抑制效应。并且，AZA+HHT可协同抑制U937（图1C）、MV4-11（图1D）和KG-1细胞的克隆形成。以上结果表明，AZA联合HHT能协同抑制AML细胞增殖、减少AML细胞克隆形成。

**图1:**
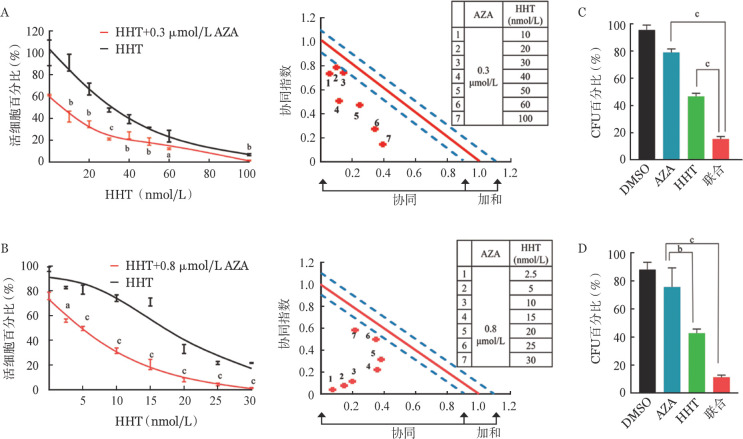
阿扎胞苷（AZA）联合高三尖杉酯碱（HHT）对急性髓系白血病细胞增殖抑制和凋亡的协同效应（实验重复3次） A AZA联合HHT对U937细胞株的增殖抑制效应； B AZA联合HHT对MV4-11细胞株的增殖抑制效应； C 0.7 µmol/L AZA联合25 nmol/L HHT作用12 h对U937细胞克隆形成的影响； D 1.5 µmol/L AZA联合15 nmol/L HHT作用12 h对MV4-11细胞克隆形成的影响。^a^*P*<0.05，^b^*P*<0.01，^c^*P*<0.001

此外，与单药相比，AZA+HHT可促进U937（*P*<0.001）（图2A）和 MV4-11（*P*<0.001）（图2B）细胞凋亡。同时，AZA+HHT增加了U937（图2C）和 MV4-11（图2D）细胞中促凋亡蛋白cleaved caspase 3和Bax蛋白表达水平，降低抗凋亡蛋白BCL2蛋白表达水平（图2C、D）。pan-caspase抑制剂Z-VAD-fmk可部分抑制AZA+HHT的细胞毒性。这些结果表明，AZA联合HHT可通过激活caspase级联反应诱导AML细胞凋亡而协同发挥细胞毒效应。

**图2:**
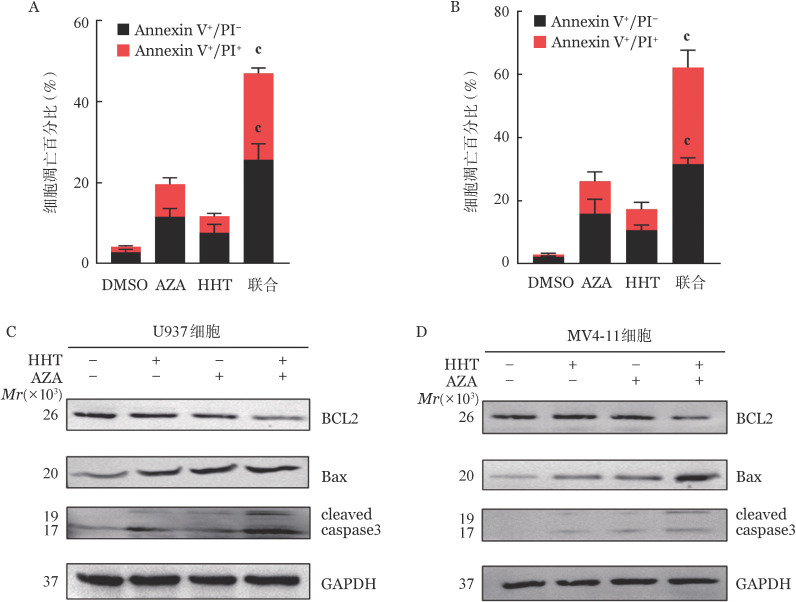
阿扎胞苷（AZA）联合高三尖杉酯碱（HHT）对急性髓系白血病细胞的促凋亡效应 A 0.7 µmol/L AZA联合25 nmol/L HHT对U937细胞的促凋亡效应； B 1.5 µmol/L AZA联合15 nmol/L HHT对MV4-11细胞的促凋亡效应； C 0.7 µmol/L AZA联合25 nmol/ L HHT处理U937细胞后凋亡蛋白的表达变化； D 1.5 µmol/L AZA联合15 nmol/L HHT处理MV4-11细胞后凋亡蛋白的表达变化。^c^*P*<0.001

2. AZA联合HHT激活AML细胞整合应激反应（ISR）信号通路：为进一步阐明AZA+HHT协同抗AML机制，分别对AZA或HHT（实验组）、DMSO（对照组）处理后U937细胞进行了全转录组测序和分析。与对照组相比，AZA或HHT处理组分别筛选出2 470和3 736个显著差异表达基因（DEGs）（|log_2_FC|>1.5，*P*<0.01）（图3A、B），取交集后筛选到1265个共同差异表达基因（图3C）；信号通路分析发现，AZA和HHT处理后共同差异表达基因主要富集于ISR信号通路，包括“应激反应内质网应激反应”、“氧化应激调节”、“饥饿反应”、“未折叠蛋白应答”、“内源性凋亡通路”等（图3D）。同时发现，联合用药处理后ISR通路相关基因及促凋亡基因ATF4、DDIT3、PUMA和PMAIP1均明显上调，而ISR通路重要调节基因c-MYC表达明显下调（图3A、B）。以上结果提示，c-MYC和ISR信号通路可能参与了AZA联合HHT协同抗AML机制。

**图3:**
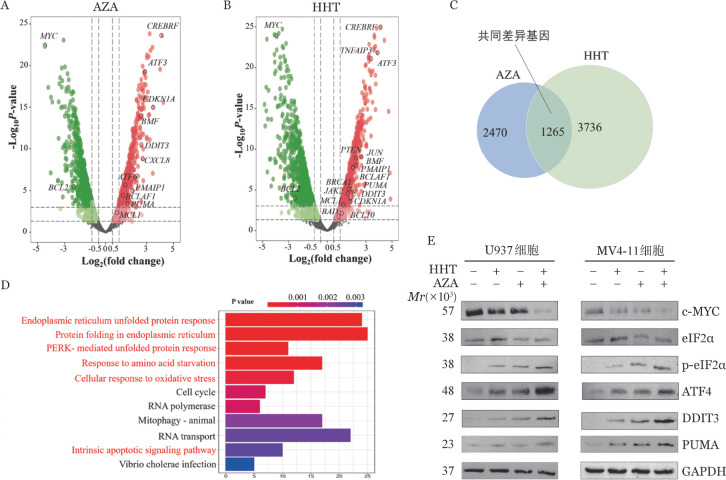
阿扎胞苷（AZA）联合高三尖杉酯碱（HHT）激活急性髓系白血病细胞整合应激反应（ISR）通路 A、B U937细胞经AZA（0.7 µmol/L）或HHT（25 nmol/L）处理4 h后进行转录组测序，火山图显示其差异表达基因（DEGs）； C AZA和HHT处理后共同差异基因的韦恩图； D AZA和 HHT处理后共同差异基因的Gene ontology（GO）通路富集（红色标注为ISR相关的信号通路）； E AZA + HHT对ISR通路的蛋白表达的影响［U937和MV4-11细胞分别经HHT（U937细胞25 nmol/L，MV4-11细胞15 nmol/L）、AZA（U937细胞0.7 µmol/L，MV4-11细胞1.5 µmol/L）单药或联合处理48 h］

RQ-PCR结果显示，AZA+HHT组中PUMA的表达水平比PMAIP1出现更明显的上调，提示PUMA的表达上调在AZA和HHT的协同效应中可能更加重要。通过Western blot法在蛋白水平研究了DMSO、AZA、HHT和AZA+HHT 4组中ISR通路中关键调节因子蛋白水平变化。结果显示，AZA+HHT组eIF2α下调，而p-eIF2α、ATF4、DDIT3和PUMA蛋白水平则上调（图3E）。并且，c-MYC蛋白水平在AZA+HHT组中下调（图3E）。以上结果提示，AZA联合HHT可激活AML细胞中ISR信号通路，ISR相关凋亡执行分子DDIT3和PUMA表达水平上升，该通路可能在AZA联合HHT协同抗AML机制中起重要作用。

3. 阻断ISR信号通路对AZA联合HHT诱导AML细胞增殖阻滞和凋亡的影响：为进一步探究ISR通路及其凋亡执行分子DDIT3和PUMA在AZA+HHT协同抗AML作用中效应，我们采用ISR通路抑制剂和靶基因敲降的方法进行进一步研究。首先将ISR信号通路抑制剂ISRIB与AML细胞进行共培养发现，在ISRIB（200 nmol/L）共培养条件下，AML细胞中AZA+HHT介导的细胞增殖抑制作用减弱（图4A），AZA+HHT介导的细胞凋亡被抑制（图4B）。此外，AZA+HHT上调了DDIT3、PUMA和cleaved caspase 3的蛋白表达水平，而ISRIB可抑制AZA+HHT联合组中上述蛋白表达的增加（图4C）。以上结果表明ISR信号通路的激活在AZA联合HHT协同机制中发挥重要作用。

**图4:**
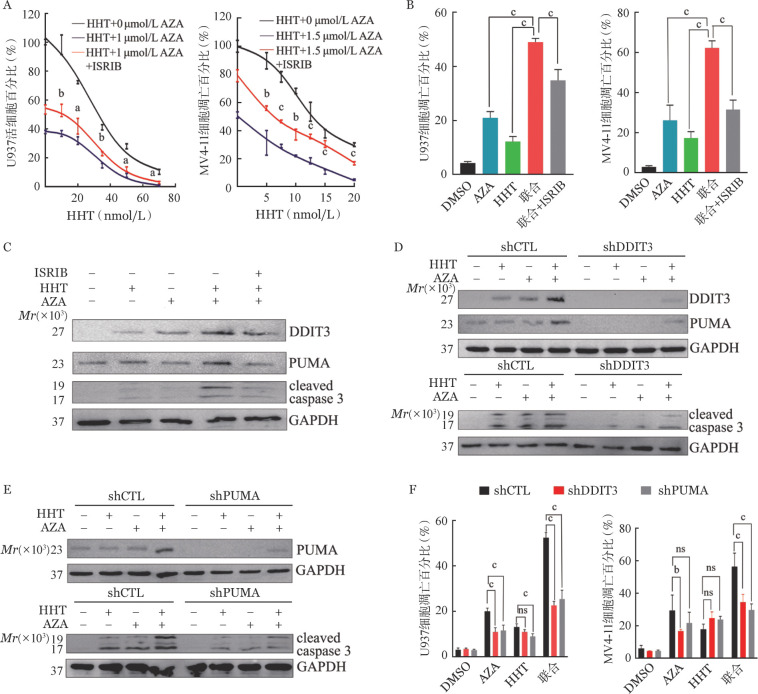
阻断ISR信号通路对阿扎胞苷（AZA）联合高三尖杉酯碱（HHT）诱导急性髓系白血病（AML）细胞增殖阻滞和凋亡的影响 A 小分子ISR抑制剂（ISRIB）对AZA联合HHT诱导细胞增殖抑制影响的剂量反应曲线； B ISRIB（200 nmol/L）对AZA联合HHT处理48 h的AML细胞凋亡的影响； C ISRIB（200 nmol/L）对AZA联合HHT处理48 h的AML细胞DDIT3、PUMA和cleaved caspase 3蛋白水平的影响； D～F 在U937细胞中敲降DDIT3（shDDIT3）或PUMA（shPUMA）对AZA联合HHT诱导的DDIT3、PUMA和cleaved caspase 3蛋白水平及凋亡的影响。shCTL：转染了空载质粒的对照；B～F中细胞处理条件如下：DMSO：对照组；AZA（U937细胞为0.7 µmol/L，MV4-11细胞为1.5 µmol/L）和（或）HHT（U937细胞为25 nmol/L，MV4-11细胞为15 nmol/L）处理48 h。ns为*P*>0.05，^a^*P*<0.05，^b^*P*<0.01，^c^*P*<0.001

我们进一步探索了DDIT3及PUMA在ISR信号传导和AZA+HHT介导凋亡中的作用。与对照（shCTL）相比，敲降DDIT3导致AZA+HHT诱导的cleaved caspase 3表达下调，并减少了AZA+HHT介导的AML细胞凋亡（图 4D、4F）。同时，敲降DDIT3可抑制AZA+HHT组PUMA蛋白水平上调（图 4D）。此外，敲降PUMA可抑制AZA+HHT组cleaved caspase 3蛋白上调（图 4E），并减少了AML细胞的凋亡（图4F）。以上结果提示，AZA联合HHT依赖ISR信号通路发挥促进AML细胞凋亡的效应，该效应是通过激活下游效应分子DDIT3和PUMA来发挥作用的。

4. c-MYC对AZA+HHT介导的ISR相关的细胞凋亡的影响：作为DDIT3的上游调控基因，c-MYC在AZA+HHT处理后表达下调。为此，我们进一步研究了c-MYC对AZA+HHT激活的ISR通路及DDIT3和PUMA表达的影响。我们运用shRNA方法敲降了AML细胞中c-MYC基因（图5A、B），发现敲降c-MYC上调了p-eIF2α蛋白的水平。同时，我们也发现在U937和MV4-11细胞中，与shCTL相比，敲降c-MYC上调了AZA+HHT组DDIT3、PUMA和cleaved caspase 3蛋白表达水平（图5C～D）。同时，敲降c-MYC增加了AZA+HHT诱导的AML（图5E）细胞凋亡。以上结果表明c-MYC是AZA+HHT介导的ISR凋亡相关通路的重要抑制调节因子。

**图5:**
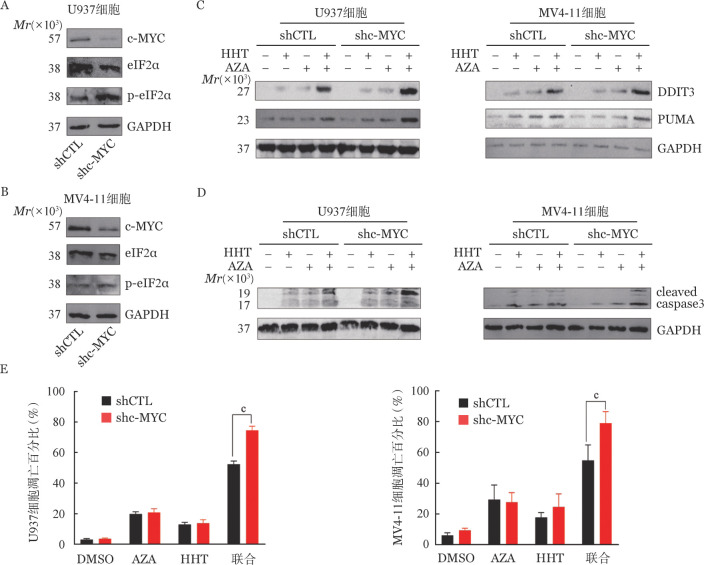
c-MYC对阿扎胞苷（AZA）联合高三尖杉酯碱（HHT）介导的ISR通路激活及细胞凋亡的影响 A、B U937和MV4-11细胞中敲降c-MYC（shc-MYC）后c-MYC蛋白的表达水平； C、D 在U937和MV4-11细胞中敲降c-MYC对经药物处理后DDIT3、PUMA和cleaved caspase 3蛋白表达水平的影响； E 敲降c-MYC对药物处理引起的细胞凋亡的影响。药物处理条件如下：DMSO：对照组；AZA（U937细胞中为0.7 µmol/L，MV4-11细胞中为1.5 µmol/L）和（或）HHT（U937细胞中为25 nmol/L，MV4-11细胞中为15 nmol/L）处理48 h。^c^*P*<0.001

## 讨论

本研究中，我们观察到AZA联合HHT对AML细胞具有协同诱导细胞凋亡、抑制细胞增殖的抗AML作用，并发现AZA+HHT通过调控c-MYC/DDIT3/PUMA 轴发挥协同抗 AML作用。

AZA能通过减少基因组甲基化和抑制mRNA的合成抑制白血病细胞增殖[8]，而HHT能够特异性地调控抑制增殖旺盛肿瘤细胞中的蛋白质生物合成[15]。因此，AZA+HHT组合可以在表观遗传、转录及蛋白质合成等多个层面上形成细胞外刺激，进而发挥协同作用效应。我们运用全转录组测序分析两药共同差异表达基因和共同作用通路的研究方法[16]–[18]，发现AZA联合HHT可引起ISR通路以及与应激相关的促凋亡基因明显上调。进一步，我们运用通路抑制剂及基因敲降手段在细胞表型、转录和蛋白水平上证实ISR通路是AZA联合HHT诱导AML细胞凋亡的关键信号通路。

ISR是真核细胞为恢复细胞稳态而启动的适应性反应，以响应各种细胞外刺激，包括低氧、葡萄糖缺乏、氨基酸缺乏、内质网中未折叠蛋白积累等[19]。eIF2α的磷酸化是ISR激活途径的共同关键步骤，它通过激活下游效应因子以进行适应性反应[14]。而当细胞应激刺激进一步加剧（强度或持续时间），p-eIF2α的下游效应因子（ATF4、DDIT3、PUMA等）最终会被激活并执行细胞程序性死亡[19]–[20]。ISRIB作为ISR信号通路的抑制剂，通过逆转eIF2α的磷酸化发挥抑制ISR信号通路的作用[21]。本研究发现，AZA+HHT处理AML细胞诱导了eIF2α磷酸化、ATF4表达，并最终增加了ISR信号途径介导的凋亡执行分子DDIT3和PUMA的表达。此外，ISR信号抑制剂、敲降DDIT3或PUMA均减弱了AZA+HHT对AML细胞的促凋亡效应。我们的研究结果表明，加重细胞外应激与触发ISR介导的凋亡密切相关[22]。

我们也观察到AZA+HHT处理后，c-MYC表达明显下调。c-MYC是AML关键致癌基因之一，与肿瘤增殖相关通路的活化密切相关，如肿瘤增殖、代谢等[23]。本研究运用敲降c-MYC的研究手段[24]–[25]，探究AZA+HHT处理后引起的ISR信号通路特异性改变。结果显示，敲降c-MYC上调了磷酸化eIF2α的水平，同时上调了DDIT3和PUMA的表达，并且增加AML细胞凋亡，与既往研究报道一致[26]–[29]。这些结果表明，c-MYC是ISR信号通路的直接抑制因子，AZA联合HHT处理引起的c-MYC下调激活并放大了ISR信号通路介导的凋亡途径。

综上，AZA联合HHT通过激活ISR信号通路，调控c-MYC/DDIT3/PUMA轴，抑制细胞增殖和促进细胞凋亡，发挥协同抗AML作用。本研究为临床应用AZA联合HHT为基础方案治疗AML的有效性提供了重要理论依据。
